# Randomized phase 3 study of lenalidomide versus chlorambucil as first-line therapy for older patients with chronic lymphocytic leukemia (the ORIGIN trial)

**DOI:** 10.1038/leu.2017.47

**Published:** 2017-02-17

**Authors:** A Chanan-Khan, M Egyed, T Robak, F A Martinelli de Oliveira, M A Echeveste, S Dolan, P Desjardins, J Z Blonski, J Mei, N Golany, J Zhang, J G Gribben

**Affiliations:** 1 Division of Hematology and Oncology, Mayo Clinic, Jacksonville, FL, USA; 2 Department of Internal Medicine, Kaposi Mor Oktato Korhaz, Kaposvar, Hungary; 3 Department of Hematology, Medical University of Lodz, Lodz, Poland; 4 Department of Oncology, Hospital Erasto Gaertner, Curitiba, Brazil; 5 Hematology Service, Hospital Universitario Donostia, San Sebastian, Spain; 6 Division of Hematology, Saint John Regional Hospital, Saint John, New Brunswick, Canada; 7 Department of Hematology, Hospital Charles LeMoyne, Greenfield Park, Quebec, Canada; 8 Celgene Corporation, Summit, NJ, USA; 9 Centre for Haemato-Oncology, Barts Cancer Institute, London, UK

Lenalidomide ((LEN) Revlimid; Celgene Corporation, Summit, NJ, USA), an oral immunomodulatory agent, demonstrated activity in phase 2 trials in patients with chronic lymphocytic leukemia (CLL) in front-line therapy^1, 2^ and in patients with relapsed and/or refractory disease.^3, 4^ An open-label, randomized, multicenter, phase 3, parallel-group study was conducted evaluating LEN as first-line therapy for elderly patients (⩾65 years) with CLL (the ORIGIN trial (NCT00910910)). Chlorambucil (CHB), a standard front-line therapy for elderly patients at trial initiation, was used as a comparator.^5, 6, 7^

The study protocol is summarized in the Supplementary Methods. Briefly, between November 2009 and March 2013, 450 CLL patients aged ⩾65 years with previously untreated, active disease and an Eastern Cooperative Oncology Group performance status (ECOG PS) score of ⩽2 were randomized (1:1) to LEN until unacceptable toxicity or progressive disease (PD), or CHB for up to 13 cycles or until unacceptable toxicity or PD. In the LEN arm, patients with creatinine clearance (CrCl) ⩾60 ml/min were given oral LEN 5 mg daily, escalated to 15 mg daily, if tolerated; those with CrCl ⩾30 to <60 ml/min were given LEN 2.5 mg daily, escalated to 7.5 mg daily, if tolerated. In the CHB arm, patients received CHB 0.8 mg/kg on days 1 and 15 of each 28-day cycle. The primary endpoint was progression-free survival (PFS). Secondary endpoints included safety, response, duration of response, time to response and overall survival (OS). The study was conducted according to good clinical practice and the ethical principles outlined in the Declaration of Helsinki. All patients provided written informed consent.

The data monitoring committee observed an imbalance in deaths between the treatment arms favoring CHB in February 2013. As a result, patients aged ⩾81 years discontinued study treatment in April 2013, and all patients receiving LEN discontinued treatment in July 2013. By March 2014, all patients in the CHB arm had also stopped treatment. Patient flow and the rationale for the data cutoff dates are provided in Supplementary Figure 1 and Supplementary Table 1, respectively.

Median age was 72 years (range 65–90) and 73 years (range 65–92) in the LEN and CHB arms, respectively. Baseline characteristics were well balanced between treatment arms (Supplementary Table 2). More patients in the LEN arm had an ECOG PS score of 0 compared with the CHB arm.

Median PFS at the February 2013 cutoff date (median follow-up 11.8 months) was 30.8 months in the LEN arm versus 23.0 months in the CHB arm. The hazard ratio (HR) for PD or death favored the CHB arm but was not statistically significant (HR 1.21; 90% confidence interval (CI) 0.88–1.66; *P*=0.323). There were 35 deaths (16.5%) in the LEN arm and 21 (9.8%) in the CHB arm. The HR for OS was 1.69 (90% CI 1.06–2.67; *P*=0.060) in favor of CHB. Similar results were seen at the April 2013 cutoff date, when all patients aged ⩾81 years discontinued treatment (Table 1). As of March 2014, 94 of the 450 patients had died (20.9%), with an equal number in each treatment arm (47/225; 20.9%). The HR for OS was 1.03 (90% CI 0.73–1.46; *P*=0.883; Supplementary Figure 2).

Overall response rate at the April 2013 cutoff date was significantly lower in the LEN arm than the CHB arm (55.1 vs 65.8% *P*=0.026). Complete response (CR) was achieved in 6 (2.7%) LEN-treated patients and 22 (9.8%) CHB-treated patients. Median time to first response was 8.9 weeks for LEN-treated patients versus 8.1 weeks for CHB-treated patients. Median duration of response had not been reached for LEN-treated patients versus 87.1 weeks for CHB-treated patients. The HR for duration of response favoring LEN was 0.74 (90% CI 0.47–1.16; *P*=0.262).

Median treatment duration at the April 2013 cutoff was shorter in the LEN arm compared with the CHB arm (207 vs 293 days, respectively), with greater frequency of treatment discontinuations due to treatment-emergent adverse events (TEAEs) in the LEN arm. Median number of treatment cycles was 7 in the LEN arm and 10 in the CHB arm. Median dose intensity was 4.9 mg per day in the LEN arm. More patients in the LEN arm than in the CHB arm had study drug interrupted and/or reduced due to adverse events (AEs; 63.8 vs 21.1%, respectively). Neutropenia was the most frequently reported TEAE, leading to dose reduction/interruption in either treatment arm. More patients in the LEN arm had dose reductions/interruptions due to neutropenia than patients in the CHB arm (31.3 vs 11.2%, respectively).

TEAEs were reported by more LEN-treated patients than CHB-treated patients (95.1 vs 90.1%, respectively; Supplementary Table 3) as were grade 3, 4 and 5 TEAEs (37.9 vs 32.3% 34.4 vs 20.6% and 9.4 vs 4.9%, respectively; Supplementary Table 4). Serious TEAEs were reported more frequently in the LEN versus CHB arm (63.8 vs 38.6%, respectively), including neutropenia (23.7 vs 14.3%), pneumonia (10.7 vs 2.7%), thrombocytopenia (8.0 vs 5.8%) and anemia (7.6 vs 4.0%). Tumor flare (serious and/or grade ⩾3) in the LEN arm tended to occur early in treatment (13 of 18 events in the first year occurred ⩽1 month of treatment), with none in the CHB arm.

TEAEs leading to treatment discontinuation were higher with LEN than CHB (29.0 vs 17.5%, respectively). The most frequently reported (⩾2%) TEAEs leading to discontinuation were thrombocytopenia, pneumonia and tumor flare for LEN and neutropenia, anemia and thrombocytopenia for CHB.

At the April 2013 cutoff date, 61 deaths had occurred—36 (16.0%) in the LEN arm and 25 (11.1%) in the CHB arm. The primary causes of death are shown in Table 2. No single cause of death appeared responsible for the higher number of deaths in the LEN arm. Most deaths occurred ⩽9 months after treatment initiation. More deaths occurred in the LEN arm within 6 months of starting treatment (6.2 vs 5.3%, respectively), and from 6–9 months following the start of treatment (4.0 vs 0.9%, respectively) compared with CHB. Thereafter, the frequency of deaths was similar for the two treatment arms. The median age of LEN-treated and CHB-treated patients who died was 74.5 years and 75.0 years, respectively.

More deaths were observed among older LEN-treated patients: in the overall LEN-treated population, 11.6% of patients were aged >80 years; however, 27.8% of those patients who died were >80 years. Of the 26 LEN-treated patients aged >80 years, 10 patients (38.5%) died compared with 26 (13.1%) of 199 LEN-treated patients aged ⩽80 years. Over-representation of deaths in older patients was not observed in the CHB arm where the proportions of patients >80 years (11.1%) and patients >80 years who died (12.0%) were similar. Fewer deaths occurred in responders than in non-responders: 10.5 vs 22.8%, respectively, in the LEN arm, and 6.1 vs 20.8%, respectively, in the CHB arm.

LEN-treated patients who died were more likely to have had baseline comorbidities requiring treatment and/or a more complex medical history than CHB-treated patients (Supplementary Table 5). A higher proportion of LEN-treated versus CHB-treated patients who died had a positive baseline Coombs test (22.2 vs 12.0%), bulky disease (lymphadenopathy >5 cm; 30.6 vs 16.0%), baseline ECOG PS of 2 (19.4 vs 4.0%) and IgG levels <700 mg/dl (47.2 vs 28.0%). These characteristics were also present in a higher proportion of LEN-treated patients who died, versus the overall LEN-treated population.

A greater proportion of patients who died had moderate renal impairment (CrCl ⩾30 to <60 ml/min). In the LEN arm, 39.1% of patients overall had moderate renal impairment, whereas of patients who died, 58.3% had moderate renal impairment. In the CHB arm, the incidence of moderate renal impairment was 43.6% overall, and 56.0% in the patients that died.

As of March 2014, a total of 20 (8.9%) patients in the LEN arm versus 32 (14.3%) in the CHB arm experienced ⩾1 second primary malignancy (SPM). A lower proportion of patients in the LEN arm experienced an invasive SPM (hematologic and solid tumor) versus the CHB arm (3.6 vs 6.3%, respectively). Hematologic SPMs occurred in 3 (1.3%) vs 4 (1.8%) patients in the LEN versus CHB arm, respectively; solid tumor SPMs occurred in 5 (2.2%) versus 10 (4.5%) patients, respectively.

At the March 2014 cutoff date, approximately one-third of patients in the LEN (36.6%) and CHB arms (33.6%) had received subsequent anticancer therapies. As there was no apparent imbalance between the treatment arms, the use of subsequent anticancer therapies was not believed to have confounded the OS results.

On the basis of these interim results of a trial that was terminated early, it appears that LEN did not prolong PFS and was associated with a lower response rate, a higher incidence of grade ⩾3 AEs and a higher number of deaths compared with CHB. Although LEN demonstrated clinical activity in a subset of patients in this trial, LEN monotherapy is not recommended as first-line therapy for patients with CLL, particularly those who are elderly and/or frail. Further research will be required to determine whether future immunomodulatory agents could have a role in the treatment of CLL.

## Figures and Tables

**Table 1:** PFS and OS by the data cutoff dates (ITT population)

*Endpoint*	*LEN*			*CHB*			*P-value*
	*Median follow-up, months*	N	*Median, months (95% CI)*	N	*Median, months (95% CI)*	*HR (90% CI)*	
*PFS*							
18 February 2013	11.8	212	30.8 (18.7, NE)	215	23.0 (19.3, 29.2)	1.21 (0.88, 1.66)	0.323
26 April 2013	12.6	225	30.8 (18.7, NE)	225	21.4 (19.3, 27.0)	1.00 (0.75, 1.34)	0.994
31 March 2014	18.8	225	30.8 (18.7, NE)	225	21.4 (19.3, 25.1)	0.99 (0.76, 1.29)	0.967

*OS*							
18 February 2013	11.8	212	NE (NE, NE)	215	NE (NE, NE)	1.69 (1.06, 2.67)	0.060
26 April 2013	12.6	225	NE (NE, NE)	225	NE (35.8, NE)	1.46 (0.95, 2.26)	0.149
31 March 2014	18.8	225	NE (NE, NE)	225	44.0 (37.3, NE)	1.03 (0.73, 1.46)	0.883

Abbreviations: CHB, chlorambucil; CI, confidence interval; HR, hazard ratio; ITT, intention-to-treat; LEN lenalidomide; NE, not estimable; OS, overall survival; PFS, progression-free survival.

**Table 2:** Investigator-assessed primary cause of death by MedDRA SOC: 26 April 2013 (ITT population)

*SOC*	n *(%) patients*	
	*LEN (*N*=225)*	*CHB (*N*=225)*
Deaths	36 (16.0)	25 (11.1)

*General disorders and administration site conditions*		
Death	4 (1.8)	3 (1.3)
Disease progression	3 (1.3)	2 (0.9)
Multi-organ failure	3 (1.3)	1 (0.4)

*Neoplasms benign, malignant and unspecified (including cysts and polyps)*		
Chronic lymphocytic leukemia	4 (1.8)	5 (2.2)
Refractory chronic lymphocytic leukemia	0 (0.0)	1 (0.4)
Hodgkin lymphoma	1 (0.4)	0 (0.0)
Metastatic small intestine carcinoma	1 (0.4)	0 (0.0)

*Cardiac disorders*		
Cardiac arrest	3 (1.3)	0 (0.0)
Cardiopulmonary failure	2 (0.9)	0 (0.0)
Cardiac failure	1 (0.4)	3 (1.3)
Pericardial effusion	1 (0.4)	0 (0.0)
Myocardial infarction	0 (0.0)	1 (0.4)

*Infections and infestations*		
Pneumonia	5 (2.2)	3 (1.3)
Respiratory tract infection	1 (0.4)	0 (0.0)
Sepsis	0 (0.0)	1 (0.4)

*Respiratory, thoracic and mediastinal disorders*		
Respiratory failure	1 (0.4)	0 (0.0)
Acute respiratory failure	0 (0.0)	1 (0.4)
Respiratory distress	0 (0.0)	1 (0.4)

*Blood and lymphatic system disorders*		
Autoimmune hemolytic anemia	1 (0.4)	1 (0.4)

*Gastrointestinal disorders*		
Lower gastrointestinal hemorrhage	1 (0.4)	0 (0.0)

*Metabolism and nutrition disorders*		
Tumor lysis syndrome	1 (0.4)	0 (0.0)

*Nervous system disorders*		
Intracranial hemorrhage	1 (0.4)	0 (0.0)

*Psychiatric disorders*		
Suicide	1 (0.4)	0 (0.0)

*Renal and urinary disorders*		
Renal failure	1 (0.4)	0 (0.0)

*Injury, poisoning and procedural complications*		
Post-procedural hemorrhage	0 (0.0)	1 (0.4)
Skull fracture	0 (0.0)	1 (0.4)

Abbreviations: CHB, chlorambucil; ITT, intention-to-treat; LEN, lenalidomide; MedDRA, Medical Dictionary for Regulatory Activities; SOC, system organ class.
