# Supplementary material for: Randomized phase 3 study of lenalidomide versus chlorambucil as first-line therapy for older patients with chronic lymphocytic leukemia (the ORIGIN trial)
Source: Leukemia. 2017 Feb 17;31(5):1240–3. doi: 10.1038/leu.2017.47 (PMC5420789; doi:10.1038/leu.2017.47)

**Supplementary Methods**

*Patient stratification*

Patient inclusion criteria included age ≥65 years, a documented diagnosis of CLL requiring treatment according to the International Workshop on Chronic Lymphocytic Leukemia (iwCLL) guidelines,^1^ and an ECOG PS of ≤2. Exclusion criteria included prior treatment for CLL, an active infection or systemic infection that had not resolved >2 months prior to initiating study treatment, pregnancy, participation in a clinical trial <28 days prior to initiating study treatment, patients testing positive for human immunodeficiency virus (HIV), hepatitis C virus or hepatitis B virus, absolute neutrophil count <1,000/μl, platelet count <50,000/μl, and presence of grade ≥2 neuropathy.

Randomization to either treatment arm was accomplished by Interactive Voice Recognition System (IVRS). Designated research personnel at investigating sites were assigned password-protected, coded identification numbers allowing access to the IVRS to enroll patients. Once the enrollment questionnaire had been completed, the IVRS assigned a patient number and study drug to the eligible patient. Patients were recommended to be randomized on day 1 of the study.

Patients were stratified at randomization by: 1) Disease stage (Rai stage I or II, or Binet stage A or B, versus Rai stage III or IV, or Binet stage C); 2) Presence of either aspartate aminotransferase/alanine aminotransferase ≥3 times the upper limit of normal, or CrCl ≥30 to <60 ml/min; and 3) Presence of one of the following poor prognostic factors: *11q* deletion, *17p* deletion, unmutated immunoglobulin heavy-chain variable region gene *(IGHV)* or β_2_­microglobulin >4.0 mg/l.

Treatment was initiated on the day of randomization. In the LEN arm, patients with normal renal function (CrCl ≥60 ml/min) were given oral LEN 5 mg daily on days 1–28. If tolerated, dose escalation to 10 mg daily was permitted in cycle 2, with escalation to 15 mg daily from cycle 3. Patients with moderate renal impairment (CrCl ≥30 to <60 ml/min) were given 2.5 mg LEN daily on days 1–28. If tolerated, escalation to 5 mg daily was permitted in cycle 2, with escalation to 7.5 mg daily from cycle 3. Patients randomized to the CHB arm were administered 0.8 mg/kg CHB (using the Broca Index to calculate weight in kg) on days 1 and 15 of each 28-day cycle for up to 13 cycles (approximately 12 months).

All patients received prophylaxis for tumor lysis syndrome, consisting of oral hydration and 300 mg/day allopurinol (100 mg/day for patients with CrCl ≥30 to <60 ml/min) for 3 days before starting treatment, and daily during the first treatment cycle. Daily tumor lysis syndrome prophylaxis was also given during the first cycle of each LEN dose escalation.

The primary endpoint of the study was PFS. Secondary endpoints included safety (type, frequency, severity of AEs and relationship of AEs to LEN); response; duration of response; time to response; and OS.

The study was carried out at 118 sites in Austria, Australia, Belgium, Brazil, Bulgaria, Canada, Chile, Columbia, Croatia, Czech Republic, Denmark, Hungary, Israel, Italy, the Netherlands, New Zealand, Poland, Portugal, Romania, Russia, South Africa, Slovakia, Spain, Serbia, the United Kingdom, and the United States of America. The protocol was reviewed by the Institutional Review Board/Independent Ethics Committee (IRB/IEC) and the study was conducted according to Good Clinical Practice and the ethical principles outlined in the Declaration of Helsinki. All patients provided written informed consent.

*Study amendments*

During the course of the study, the study design was altered according to two protocol amendments. The first of these, effective April 2013, and based on the recommendations of the data monitoring committee, directed all patients aged ≥81 years to be immediately discontinued from either treatment. The second amendment, effective July 2013, directed all patients receiving LEN to immediately discontinue treatment. This was based on a February 18, 2013 data cut-off, which revealed an imbalance of deaths in the LEN arm and a trend in OS favoring CHB treatment. These findings led to the study being placed on clinical hold by the US Food and Drug Administration (FDA). Patients affected by the first protocol amendment remained in the study, undergoing protocol-specified follow-up assessments every 28 days, and were assessed for survival every 4 months following the onset of PD. Following the second protocol amendment, all patients receiving LEN immediately discontinued treatment, but patients in the CHB arm could continue to receive CHB for the protocol-specified duration. Irrespective of treatment arm, patients who discontinued treatment entered the survival follow-up period, which included monitoring every 4 months for OS, SPMs, and other CLL therapies for at least 5 years after the last patient was randomized.

*Data cut-off points*

Three key clinical data cut-off dates for this trial are reported herein (Supplementary Figure 1, Supplementary Table 1). The first is February 18, 2013, the data cut-off that formed the basis of the FDA’s clinical hold. The second is the April 26, 2013 data cut-off, when the first patient discontinued treatment as a result of the first study amendment, which directed all patients aged ≥81 years to immediately discontinue either treatment. Finally, the March 31, 2014 data cut-off is the date when all patients in the CHB arm had stopped treatment.

The analyses based on the March 31, 2014 data cut-off are confounded by the early study termination of patients aged ≥81 years and the treatment discontinuation for patients in the LEN arm due to the amendments. Therefore, the analyses based on the April 26, 2013 data cut-off provide the most valid comparison between the two treatment arms. However, analyses of PFS and OS based on the March 31, 2014 data cut-off provide longer follow-up duration.

All randomized patients who received the study drug were included in the safety analyses. The cut-off date used in the reported safety analyses was April 26, 2013, with the exception of SPM data, which were based on the March 31, 2014 cut-off date with the longer follow-up.

*Statistical analyses*

A total of 450 patients were randomized (1:1) to each treatment arm in order to detect a 40% improvement in median PFS in the LEN group versus the CHB group with 80% statistical power.

Continuous variables were summarized using descriptive statistics, and categorical variables were summarized using frequency tabulations for each treatment and for both treatments combined.

Efficacy analyses were performed on the intention-to-treat (ITT) population, which included all randomized patients. PFS was calculated as the time from randomization to the first documented disease progression, as confirmed by investigator assessment or death due to any cause (whichever occurred first). In each analysis, PFS was analyzed using the Kaplan–Meier method and log-rank test. Responses were investigator-assessed using the iwCLL guidelines for the diagnosis and treatment of CLL.^1^ Exact test procedures for proportions were used to compare response rates between the two treatment arms.

**Reference**

1. Hallek M, Cheson BD, Catovsky D, Caligaris-Cappio F, [Dighiero G](http://www.ncbi.nlm.nih.gov/pubmed/?term=Dighiero%20G%5BAuthor%5D&cauthor=true&cauthor_uid=18216293), [Döhner H](http://www.ncbi.nlm.nih.gov/pubmed/?term=D%C3%B6hner%20H%5BAuthor%5D&cauthor=true&cauthor_uid=18216293) *et al*. Guidelines for the diagnosis and treatment of chronic lymphocytic leukemia: a report from the International Workshop on Chronic Lymphocytic Leukemia updating the National Cancer Institute-Working Group (NCI-WG) 1996 Guidelines. *Blood* 2008 **111**:5446–5456.

**Supplementary Tables**

| **Supplementary Table 1** Rationale for clinical cut-off dates | |
| --- | --- |
| *Clinical cut-off date* | *Rationale for the cut-off date* |
| February 18, 2013 | Basis of the FDA clinical hold |
| April 26, 2013^a^ | The first patient discontinued treatment as a result of the first protocol amendment (all patients aged ≥81 years discontinued from treatment in both treatment arms) |
| March 31, 2014^b^ | The last patient discontinued CHB treatment |

Abbreviations: CHB, chlorambucil; FDA, US Food and Drug Administration; LEN, lenalidomide; OS, overall survival; PFS, progression-free survival.
*^a^*Data from this cut-off date provide the most valid comparison between the two treatment arms. However, for PFS and OS, the duration of follow-up was relatively short.  ^b^Data from this cut-off date are confounded by the protocol amendments that mandated the early termination of treatment of patients age ≥81 years, and by subsequent treatment discontinuation of patients in the LEN arm.

| **Supplementary Table 2.** Baseline demographic and clinical characteristics, and laboratory data by treatment group | | |
| --- | --- | --- |
| *Characteristic* |  | |
|  | *LEN (N = 225)* | *CHB (N = 225)* |
| Age (years) |  |  |
| Mean | 73.0 | 73.3 |
| Standard deviation | 5.72 | 5.72 |
| Median | 72 | 73 |
| Min, max | 65.0, 90.0 | 65.0, 92.0 |
| Age distribution, n (%) |  |  |
| <70 years | 66 (29.3) | 65 (28.9) |
| ≥70 years | 159 (70.7) | 160 (71.1) |
| >80 years | 26 (11.6) | 25 (11.1) |
| Sex, n (%) |  |  |
| Male | 132 (58.7) | 142 (63.1) |
| Female | 93 (41.3) | 83 (36.9) |
| Race, n (%) |  |  |
| White or Caucasian | 216 (96.0) | 213 (94.7) |
| Black or African American | 1 (0.4) | 6 (2.7) |
| Asian | 0 (0.0) | 1 (0.4) |
| Other | 8 (3.6) | 5 (2.2) |
| β_2_-microglobulin >4.0 mg/l, n (%) |  |  |
| Yes | 120 (53.3) | 112 (49.8) |
| No | 102 (45.3) | 110 (48.9) |
| NA | 3 (1.3) | 3 (1.3) |
| *11q* deletion, n (%) |  |  |
| Yes | 43 (19.1) | 42 (18.7) |
| No | 173 (76.9) | 177 (78.7) |
| Missing | 9 (4.0) | 6 (2.7) |
| *13q* deletion, n (%) |  |  |
| Yes | 124 (55.1) | 124 (55.1) |
| No | 92 (40.9) | 95 (42.2) |
| Missing | 9 (4.0) | 6 (2.7) |
| *17p* deletion, n (%) |  |  |
| Yes | 19 (8.4) | 16 (7.1) |
| No | 201 (89.3) | 205 (91.1) |
| Missing | 5 (2.2) | 4 (1.8) |
| *IGHV* mutational status, n (%) |  |  |
| *IGHV* mutated | 89 (39.6) | 79 (35.1) |
| *IGHV* unmutated | 123 (54.7) | 130 (57.8) |
| Missing | 13 (5.8) | 16 (7.1) |
| ZAP-70, n (%) |  |  |
| Negative | 128 (56.9) | 124 (55.1) |
| Positive | 84 (37.3) | 91 (40.4) |
| Missing | 13 (5.8) | 10 (4.4) |
| Presence of at least 1 poor prognostic factor, n (%) |  |  |
| Yes | 176 (78.2) | 171 (76.0) |
| No | 40 (17.8) | 45 (20.0) |
| Unknown | 9 (4.0) | 9 (4.0) |
| Clinical staging, n (%) |  |  |
| Binet A | 25 (11.1) | 29 (12.9) |
| Binet B | 48 (21.3) | 44 (19.6) |
| Binet C | 22 (9.8) | 24 (10.7) |
| Rai I | 22 (9.8) | 26 (11.6) |
| Rai II | 50 (22.2) | 42 (18.7) |
| Rai III | 34 (15.1) | 41 (18.2) |
| Rai IV | 24 (10.7) | 18 (8.0) |
| Missing | 0 | 1 (0.4) |
| Disease stage, n (%) |  |  |
| Rai stage I or II or Binet stage A or B | 145 (64.4) | 141 (62.7) |
| Rai stage III or IV or Binet stage C | 80 (35.6) | 83 (36.9) |
| Missing | 0 | 1 (0.4) |
| Bulky disease (lymphadenopathy >5 cm), n (%) |  |  |
| Yes | 53 (23.6) | 59 (26.2) |
| No | 144 (64.0) | 138 (61.3) |
| Missing | 28 (12.4) | 28 (12.4) |
| Total area of measurable lymph nodes (SPD) from imaging, n (%) |  |  |
| N | 224 | 223 |
| ≤25th percentile | 44 (19.6) | 48 (21.5) |
| >25th to 50th percentile | 53 (23.7) | 39 (17.5) |
| >50th to 75th percentile | 40 (17.9) | 52 (23.3) |
| >75th percentile | 42 (18.8) | 49 (22.0) |
| Missing | 45 (20.1) | 35 (15.7) |
| ECOG PS score, n (%) |  |  |
| 0 | 110 (48.9) | 94 (41.8) |
| 1 | 91 (40.4) | 111 (49.3) |
| 2 | 24 (10.7) | 20 (8.9) |
| Calculated CrCl, n (%) |  |  |
| ≥30 to <60 mL/min | 88 (39.1) | 98 (43.6) |
| ≥60 mL/min | 135 (60.0) | 124 (55.1) |
| Missing | 2 (0.9) | 3 (1.3) |
| Baseline comorbidity status,^a^ n (%) |  |  |
| ≤2 SOC2 | 176 (78.2) | 174 (77.3) |
| >2 SOC2^b^ | 49 (21.8) | 51 (22.7) |

Abbreviations: CHB, chlorambucil; CIRS, Cumulative Illness Rating Scale; CrCl, creatinine clearance; ECOG, Eastern Cooperative Oncology Group; LEN, lenalidomide; NA, not applicable; PS, performance status; SPD, sum of the products of the greatest perpendicular diameters; SOC, system organ class.
^a^Comorbidity identified as a medical history of SOC that was affected at baseline and the patient was actively being treated for the condition. ^b^Equivalent to a minimum 6 points on the CIRS.

| **Supplementary Table 3.** TEAEs reported by ≥10% of patients in either treatment arm: April 26, 2013 data cut-off (safety population) | | |
| --- | --- | --- |
| *SOC^a^* | *n (%) patients* | |
|  | *LEN (N = 224)* | *CHB (N = 223)* |
|  |  |  |
| Patients with at least 1 TEAE^b^ | 213 (95.1) | 201 (90.1) |
| Blood and lymphatic system disorders |  |  |
| Neutropenia | 124 (55.4) | 83 (37.2) |
| Anemia | 77 (34.4) | 47 (21.1) |
| Thrombocytopenia | 74 (33.0) | 53 (23.8) |
| Infections and infestations |  |  |
| Pneumonia | 32 (14.3) | 7 (3.1) |
| General disorders and administration site conditions |  |  |
| Fatigue | 63 (28.1) | 52 (23.3) |
| Pyrexia | 41 (18.3) | 21 (9.4) |
| Peripheral edema | 40 (17.9) | 16 (7.2) |
| Gastrointestinal disorders |  |  |
| Diarrhea | 63 (28.1) | 30 (13.5) |
| Nausea | 32 (14.3) | 61 (27.4) |
| Constipation | 28 (12.5) | 16 (7.2) |
| Abdominal pain | 27 (12.1) | 11 (4.9) |
| Vomiting | 10 (4.5) | 29 (13.0) |
| Skin and subcutaneous tissue disorders |  |  |
| Rash | 39 (17.4) | 20 (9.0) |
| Neoplasms benign, malignant and unspecified (including cysts and polyps) |  |  |
| Tumor flare^c^ | 88 (39.3) | 11 (4.9) |
| Investigations |  |  |
| Weight decreased | 33 (14.7) | 21 (9.4) |
| Musculoskeletal and connective tissue disorders |  |  |
| Back pain | 28 (12.5) | 17 (7.6) |
| Metabolism and nutrition disorders |  |  |
| Decreased appetite | 30 (13.4) | 13 (5.8) |
| Respiratory, thoracic and mediastinal disorders |  |  |
| Cough | 37 (16.5) | 19 (8.5) |

Abbreviations: AE, adverse event; CHB, chlorambucil; LEN, lenalidomide; SOC, system organ class; TEAE, treatment-emergent adverse event. ^a^SOC and preferred terms are coded using the MedDRA version 16.1. ^b^TEAE defined as any AE that occurred/worsened on or after the first treatment with study drug and within 30 days after treatment phase end-date; a patient with multiple occurrences of a TEAE is counted only once in that TEAE category.  ^c^Grade 1 or 2 tumor flare in the LEN arm n = 76, in the CHB arm, n = 11; Grade ≥3 tumor flare in the LEN arm n = 12, and none in the CHB arm.

| **Supplementary Table 4.** Grade 3, 4, or 5 TEAEs occurring in ≥2% of patients, by treatment: April 26, 2013 data cut-off (safety population) | | | | | | | | | |
| --- | --- | --- | --- | --- | --- | --- | --- | --- | --- |
| *SOC^a^* | *LEN (N = 224), n (%)* | | | | *CHB (N = 223), n (%)* | | | | |
|  | *Grade 3* | *Grade 4* | *Grade 5* | *Total* | | *Grade 3* | *Grade 4* | *Grade 5* | *Total* |
| Patients with at least 1 Grade 3, 4, or 5 TEAE^b^ | 85 (37.9) | 77 (34.4) | 21 (9.4) | 183 (81.7) | | 72 (32.3) | 46 (20.6) | 11 (4.9) | 129 (57.8) |
| Blood and lymphatic system disorders |  |  |  |  | |  |  |  |  |
| Neutropenia | 53 (23.7) | 57 (25.4) | 0 (0.0) | 110 (49.1) | | 40 (17.9) | 33 (14.8) | 0 (0.0) | 73 (32.7) |
| Thrombocytopenia | 34 (15.2) | 21 (9.4) | 0 (0.0) | 55 (24.6) | | 23 (10.3) | 14 (6.3) | 1 (0.4) | 38 (17.0) |
| Anemia | 11 (4.9) | 4 (1.8) | 0 (0.0) | 15 (6.7) | | 6 (2.7) | 3 (1.3) | 0 (0.0) | 9 (4.0) |
| Febrile neutropenia | 6 (2.7) | 3 (1.3) | 0 (0.0) | 9 (4.0) | | 5 (2.2) | 0 (0.0) | 0 (0.0) | 5 (2.2) |
| Infections and infestations |  |  |  |  | |  |  |  |  |
| Pneumonia | 12 (5.4) | 4 (1.8) | 5 (2.2) | 21 (9.4) | | 1 (0.4) | 1 (0.4) | 3 (1.3) | 5 (2.2) |
| Metabolism and nutrition disorders |  |  |  |  | | 7 (3.1) | 4 (1.8) | 0 (0.0) | 11 (4.9) |
| Hyperkalemia | 6 (2.7) | 0 (0.0) | 0 (0.0) | 6 (2.7) | | 1 (0.4) | 0 (0.0) | 0 (0.0) | 1 (0.4) |
| Hyponatremia | 6 (2.7) | 0 (0.0) | 0 (0.0) | 6 (2.7) | | 1 (0.4) | 2 (0.9) | 0 (0.0) | 3 (1.3) |
| Dehydration | 5 (2.2) | 0 (0.0) | 0 (0.0) | 5 (2.2) | | 1 (0.4) | 0 (0.0) | 0 (0.0) | 1 (0.4) |
| Skin and subcutaneous tissue disorders |  |  |  |  | |  |  |  |  |
| Rash | 11 (4.9) | 1 (0.4) | 0 (0.0) | 12 (5.4) | | 1 (0.4) | 0 (0.0) | 0 (0.0) | 1 (0.4) |
| General disorders and administration site conditions |  |  |  |  | |  |  |  |  |
| Fatigue | 12 (5.4) | 0 (0.0) | 0 (0.0) | 12 (5.4) | | 13 (5.8) | 0 (0.0) | 0 (0.0) | 13 (5.8) |
| Asthenia | 6 (2.7) | 1 (0.4) | 0 (0.0) | 7 (3.1) | | 3 (1.3) | 0 (0.0) | 0 (0.0) | 3 (1.3) |
| Neoplasms benign, malignant and unspecified (including cysts and polyps) |  |  |  |  | |  |  |  |  |
| Tumor flare | 12 (5.4) | 0 (0.0) | 0 (0.0) | 12 (5.4) | | 0 (0.0) | 0 (0.0) | 0 (0.0) | 0 (0.0) |

Abbreviations: AE, adverse event; CHB, chlorambucil; LEN, lenalidomide; SOC, system organ class; TEAE, treatment-emergent adverse event.
^a^MedDRA version 16.1: listed in descending order based on frequencies of combined Grade 3–5 TEAEs in LEN arm. ^b^TEAE defined as any AE that occurred/worsened on or after the first treatment of study drug and within 30 days after treatment phase end-date; a patient with multiple occurrences of a TEAE is counted only once in that TEAE category.

| **Supplementary Table 5.** Medical history of interest for patients who died before April 26, 2013 (safety population) | | |
| --- | --- | --- |
| *Medical history^a^* | *Patients, n (%)* | |
|  | *LEN (N = 35)* | *CHB (N = 25)* |
| Hypertension | 18 (51.4) | 14 (56.0) |
| Infection | 9 (25.7) | 3 (12.0) |
| Myocardial infarction/ ischemic heart disease | 5 (14.3) | 5 (20.0) |
| Cardiac arrhythmias | 4 (11.4) | 3 (12.0) |
| Renal failure | 4 (11.4) | 0 (0.0) |
| Respiratory infections | 4 (11.4) | 2 (8.0) |
| VTE events | 4 (11.4) | 1 (4.0) |
| Hepatic disorders | 3 (8.6) | 0 (0.0) |
| Thrombocytopenia | 2 (5.7) | 0 (0.0) |
| Bleeding | 2 (5.7) | 0 (0.0) |
| Autoimmune hemolytic anemia | 2 (5.7) | 1 (4.0) |
| ATE events | 2 (5.7) | 1 (4.0) |
| Pneumonia | 1 (2.9) | 0 (0.0) |
| Rash | 1 (2.9) | 0 (0.0) |
| Respiratory disorders | 1 (2.9) | 0 (0.0) |
| Interstitial lung disease | 1 (2.9) | 0 (0.0) |
| Cardiac failure | 0 (0.0) | 2 (8.0) |

Abbreviations: ATE, arterial thromboembolic; CHB, chlorambucil; LEN, lenalidomide; SOC, system organ class; VTE, venous thromboembolic.
^a^Due to overlap in the search criteria used for some of the medical history categories, some SOCs are included in more than one medical history category.

**Supplementary Figures**

**Supplementary Figure 1.** CONSORT diagram with the interim data analyses. Abbreviations: CHB, chlorambucil; LEN, lenalidomide**.**

*23 patients were randomized after the first data cut-off on February 18, 2013 (13 in the LEN arm and 10 in the CHB arm).
^†^The last patient discontinued CHB treatment on March 31, 2014.

**Supplementary Figure 2.** Kaplan–Meier plot of OS: March 31, 2014 data cut-off. Abbreviations: CHB, chlorambucil; CI, confidence interval; HR, hazard ratio; LEN lenalidomide; OS, overall survival.

**

Supplementary Figure 1.**

**Supplementary Figure 2.**


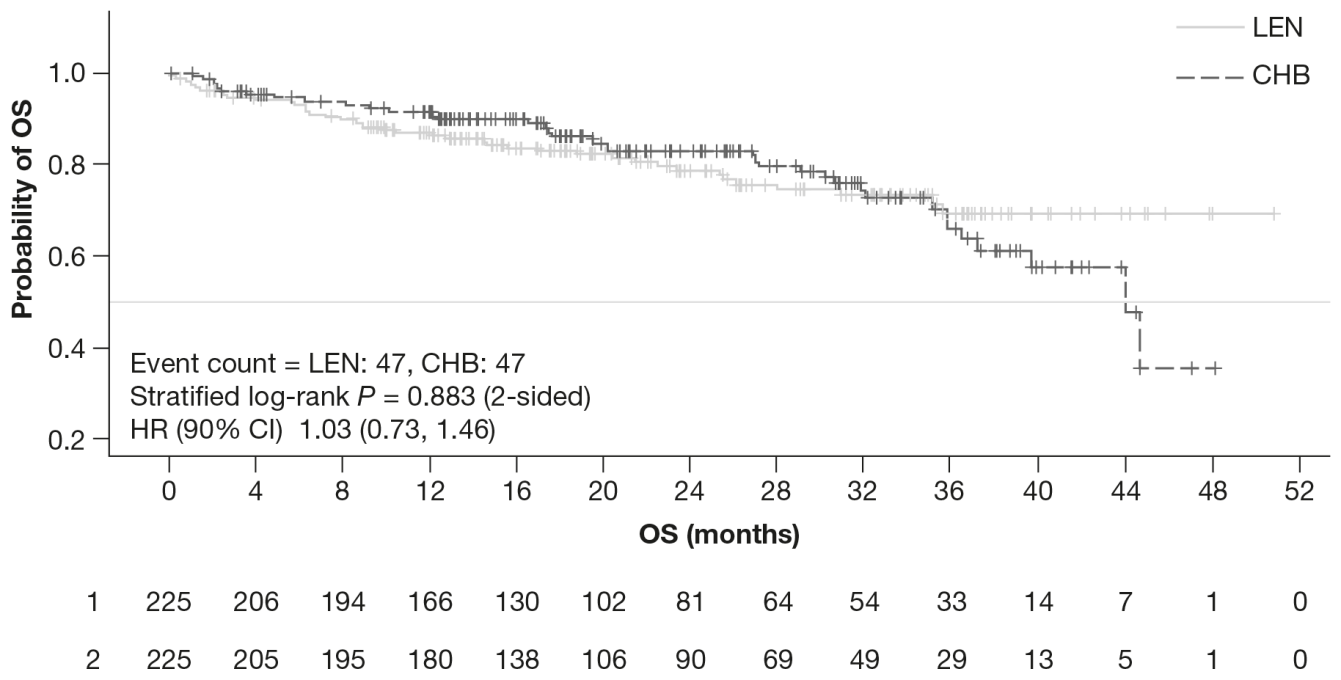

Supplement: Supplementary Information [file leu201747x1.docx]
